# Pneumococcal Meningitis in an Adolescent with Fever and Foot Ache

**DOI:** 10.1155/2013/516746

**Published:** 2013-07-14

**Authors:** Catarina Dias, Cláudia Pedrosa, Jorge Romariz, Mafalda Santos, Lúcia Rodrigues

**Affiliations:** ^1^Paediatrics Department, Centro Hospitalar de Vila Nova de Gaia/Espinho, EPE, Unidade II, 4400-129 Vila Nova de Gaia, Portugal; ^2^Orthopaedics Department, Centro Hospitalar de Vila Nova de Gaia/Espinho, EPE, Unidade II, 4400-129 Vila Nova de Gaia, Portugal

## Abstract

Invasive pneumococcal disease predominantly affects younger children, elderly, and immunocompromised patients. Pneumococcal meningitis is a particularly important form of presentation, considering its high rate of morbimortality. We present the case of a previously healthy 12-year-old adolescent male who was hospitalized due to suspicion of osteoarticular infection in his left foot. A few hours later, he developed meningeal signs, exhibiting slight pleocytosis and *Streptococcus pneumoniae* isolates in both cerebrospinal fluid and blood. Imaging studies were inconclusive regarding the nature of the foot disorder. We considered the hypothesis of osteomyelitis of the navicular bone as the most likely, for which he completed six weeks of antibiotic therapy. There was a favorable clinical evolution, along with complete absence of osteoarticular or neurological sequelae. The relevance of this clinical case resides in the unusual presentation of invasive pneumococcal disease in this age group, as well as in the rare form of orthopedic involvement.

## 1. Introduction

Invasive pneumococcal disease (IPD) is an infectious illness defined by the isolation of *Streptococcus pneumoniae *from a normally sterile site. It usually presents as bacteraemia, meningitis, or pneumonia, although virtually any site can be involved [1]. Bacteraemia and meningitis are the most common manifestations of IPD in children younger than 2 years, while older children and adults most often develop pneumococcal pneumonia. Uncommon presentations include peritonitis, arthritis, osteomyelitis, endocarditis, and pericarditis. These localized infections can occur simultaneously or following an episode of pneumococcal bacteraemia [2].

The incidence of IPD in Portugal is not officially known, as it is not a mandatory notification disease. A multicenter surveillance study has been initiated, in 2004, by the Portuguese Pediatrics Society [3]. According to their last report, IPD had a global incidence of 18.6 : 100,000 children between 2008 and 2010; those younger than 5 years accounted for over 75% of the cases. Meningitis was the third most common clinical presentation (14.1%), following pneumonia (51.5%) and bacteraemia (18.0%). 

In developed countries, *Streptococcus pneumoniae *is currently the most frequent cause of bacterial meningitis beyond the neonatal period, being responsible for numerous neurological sequelae among surviving patients [2, 4]. 

## 2. Clinical Case

A 12-year-old adolescent male presented to the Emergency Department, in January 2010, with a febrile illness. For the last 3 days, he had been complaining of dry cough, diffuse myalgia, frontal headache, and sporadic postprandial vomiting. Two days before admission he developed intermittent high fever (axillary temperature 40°C), cervicalgia, and a progressively worsening left midfoot ache. He denied any trauma history or previous limping or pain. His personal medical history was unremarkable and he had received all the vaccines included in the National Plan, which encloses meningococcal serogroup C and* Haemophilus influenzae *type b immunizations but not an antipneumococcal vaccine.

On physical examination, he was alert and oriented, with normal vital signs. He had an abnormal gait, avoiding left plantar pressure. The anterior surface of his left ankle was tender, swollen, and warm to touch. Clinical examination was otherwise unremarkable, including normal breath sounds and absence of meningeal signs. Laboratory evaluation revealed a mildly elevated leukocyte count (13,540/*μ*L) with neutrophilia (11,860/*μ*L), as well as elevated C-reactive protein (14 mg/dL) and erythrocyte sedimentation rate (55 mm/h). There were no abnormalities on thorax, left foot, and ankle radiographs.

Considering the diagnostic hypothesis of osteoarticular infection, the patient was then admitted for further investigation. A few hours later, he presented worsening neck pain, fever, and neck stiffness. Lumbar puncture revealed clear and colourless cerebrospinal fluid (CSF), with mild pleocytosis (23/*μ*L leukocytes, predominantly polymorphonuclear—20 of 23) and normal erythrocytes (7/*μ*L), glucose (77 mg/dL), and protein (28.5 mg/dL) levels. Empirical antibiotic therapy with ceftriaxone and vancomycin was initiated. Pneumococcus was identified by CSF Gram-stain and latex particle agglutination tests. The same agent was later isolated from blood culture—a penicillin-susceptible *Streptococcus pneumoniae*, serotype 1.

**Figure 1:**
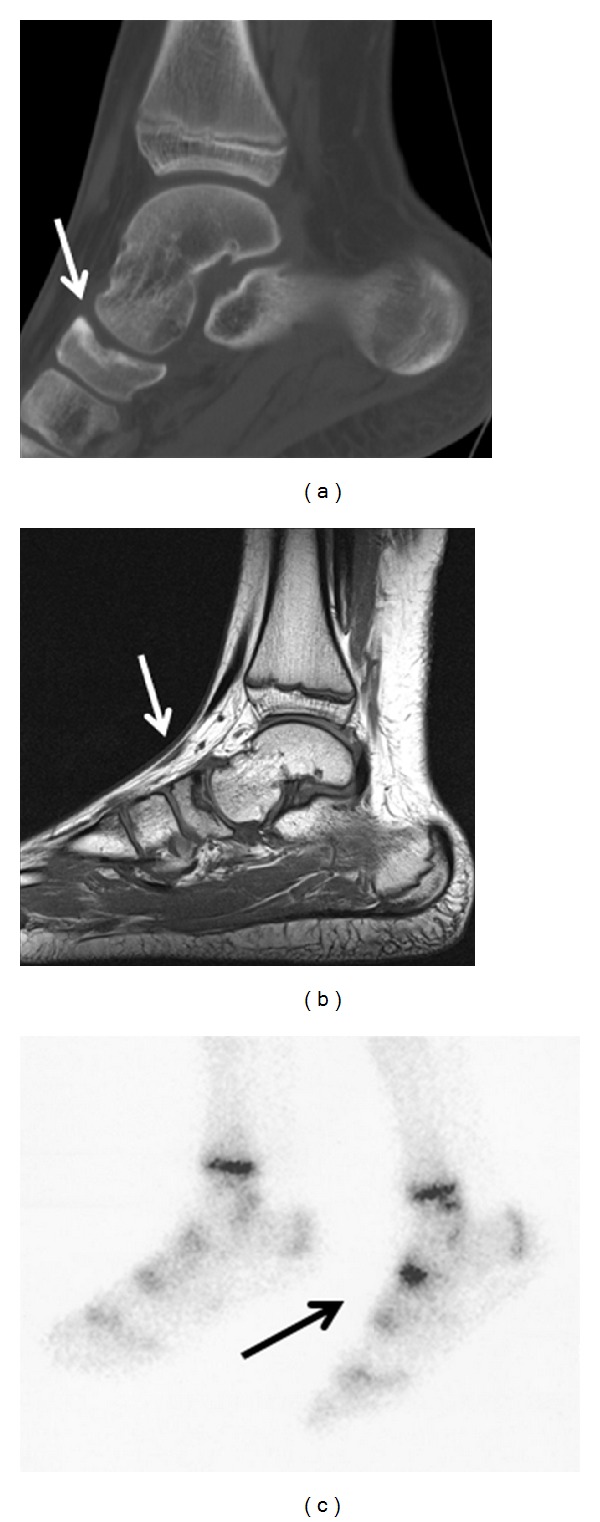
Left foot (a) CT scan: navicular bone indentation and subcortical lesion (arrow). (b) MRI: talonavicular joint effusion and irregularity of the proximal articular surface of the navicular bone (arrow). (c) Osseous scintigraphy: hyperfixation focus at the left navicular bone (arrow).

An articular computerized tomography (CT) scan of the patient's left ankle disclosed a navicular bone indentation with subcortical lesion, but it could not differentiate whether it was the result of an osteochondritis dissecans or of an osteomyelitis (Figure 1(a)). A magnetic resonance imaging (MRI) of the same region revealed a talonavicular joint effusion and irregularity of the proximal articular surface of the navicular bone, with a small cortical depression, suggesting an osteochondritis dissecans (Figure 1(b)). Osseous scintigraphy brought out intense hyperfixation at the left navicular bone (Figure 1(c)).

Since the hypothesis of navicular osteomyelitis could not be excluded, the patient was given intravenous ceftriaxone for 2 weeks, followed by 4 weeks of an oral third-generation cephalosporin. Bed rest was encouraged during the first week of treatment.

**Figure 2:**
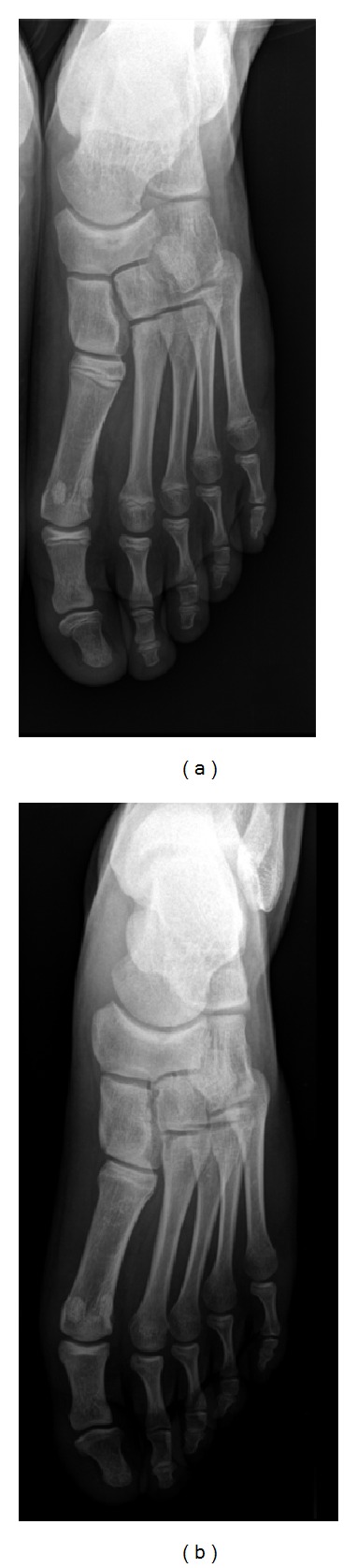
Radiographs of the left foot at 4-month (a) and 2-year (b) follow-up consultations, showing a favorable radiologic evolution.

There was a favorable clinical evolution, with sustained apyrexia after the first 3 days of antibiotherapy and progressive improvement of inflammatory signs. By the end of the treatment, the patient had resumed normal mobility, denying any osteoarticular symptoms, and 3 months later he was allowed to practice sports. After a 2-year follow-up he remains asymptomatic and presents normal osteoarticular, neurological, and audiologic examinations. Radiographs of the left foot corroborate the positive clinical outcome (Figure 2). Basic laboratory evaluation has not disclosed any immunodeficiency. 

## 3. Discussion

Osteochondritis dissecans (OCD), despite its misleading designation, is a noninflammatory pathologic process characterized by focal subchondral bone necrosis and the eventual release of bony fragments into the joint space. Repetitive trauma is often implicated in a probably multifactorial etiology. It is a rare disorder in both children and adults, predominantly affecting males aged from 10 to 20. Most cases of OCD occur in the knee, but other joints can be affected [5, 6].

Clinical presentation varies from subtle discomfort to local tenderness, swelling, and functional limitation, though asymptomatic lesions have also been described. On conventional radiographs, osteochondral lesions may appear normal; CT scans may reveal a cortical depression or loose bony fragment; scintigraphic findings are nonspecific, demonstrating a mild-to-marked increase in focal uptake; MRI can detect radiographically occult lesions, including joint effusion and soft tissue lesions not evident on CT scans, being the most accurate method for staging lesions and guiding clinical management [7].

Some authors consider two separate OCD entities—the juvenile form, occurring in patients with open physes, and the adult form, which manifests after growth plate closure. Younger patients with stable lesions have the best prognosis. In this age group, ODC has several characteristics in common with the osteochondroses and could represent an advanced stage of these conditions [8].

Only a few cases of OCD of the tarsal navicular have been described in the literature, as well as a limited number of navicular osteochondrosis, also known as Kohler's disease [9]. Kohler's disease is usually unilateral and most often affects boys. Clinical onset generally happens between the ages of 2 and 10, with midtarsal pain and radiographic changes—increased radiodensity, rarefaction, or fragmentation and eventual narrowing of the navicular bone. Its pathophysiology is best explained by mechanical pressure associated with a delayed ossification—navicular is the last tarsal bone to ossify and can get compressed between the already ossified talus and cuneiforms, thus, suffering ischemia and necrosis. Recovery is usually complete and radiographic findings may be normal 6 to 18 months after onset. 

Our patient's imagiologic findings were compatible with the diagnosis of OCD, in its juvenile form. However, he denied previous midfoot tenderness or pain with weight bearing. The sudden onset of symptoms, in association with the presence of local heat, suggests an acute inflammatory process. Therapeutic decision contemplated a possible osteomyelitis in the context of pneumococcal bacteraemia. Whether the patient was already facing an asymptomatic OCD by the time of acute infection, and whether or not this worked as a weak spot for pneumococcal invasion, we could not ascertain.

Osteomyelitis due to *S. pneumoniae* has been rarely recognized, despite pneumococcal known virulence. Isolated case reports describe patients with underlying conditions or at the extremes of age. Pneumococcal meningitis, while being a more common manifestation of IPD, is also a rare event among healthy adolescents—even when not specifically immunized.

The epidemiology of IPD has been greatly changed by pneumococcal conjugate vaccines [10]. In our country, the commercialization of the 7-valent vaccine was introduced in 2001, followed by the 13-valent vaccine in 2010. Between 2006 and 2008, there was a perceived emergence of some nonvaccine serotypes, most notably of serotypes 1 and 19A; serotype 1 occurred mostly in children older than 5 years (66.7%), being responsible for pneumonia in the majority of cases (87.5%) [3, 11]. 

The clinical manifestations of pneumococcal serotype 1 infection in our patient were quite atypical, and, fortunately, the prognosis was less severe than what could be expected. In a study of pneumococcal meningitis in Denmark, serotype 1 was associated with a much lower case-fatality rate than serotype 3 (3 versus 23%) [12]. On the contrary, an outbreak of pneumococcal serotype 1 meningitis in Northern Ghana presented a case-fatality rate of 44,4%. This was shown to be related to a hypervirulent clonal complex [13].

Among adult patients, the association of pneumococcal meningitis and osteoarticular infection seems to be a somewhat common event. Weisfelt et al. isolated *S. pneumoniae* from CSF of 23% of the patients with community-acquired meningitis and arthritis. Predisposing factors, early-onset arthritis, and monoarticular involvement, most frequently found in patients with pneumococcal meningitis, suggested an infectious process, instead of the immune-mediated arthritis often seen following meningococcal meningitis [14].

In this case report, we emphasize the rare presentation of invasive pneumococcal disease in a healthy adolescent—through bacteraemia with meningitis—as well as the difficulty in defining the accompanying orthopedic pathology, which was the primary cause of hospital admission.
